# White Wine Antioxidant Metabolome: Definition and Dynamic Behavior during Aging on Lees in Oak Barrels

**DOI:** 10.3390/antiox12020395

**Published:** 2023-02-06

**Authors:** Rémy Romanet, Régis D. Gougeon, Maria Nikolantonaki

**Affiliations:** Institut Agro Dijon, Institut Universitaire de la Vigne et du Vin, UMR PAM Université de Bourgogne Franche-Comté, Jules Guyot, F-21000 Dijon, France

**Keywords:** peptides, oxidation, chardonnay, nucleophilic compounds, metabolomics

## Abstract

White wines’ oxidative stability is related to a flow of chemical reactions involving a number of native wine compounds comprising their antioxidant metabolome. By applying the combination of powerful and modern analytical approaches (EPR, DPPH, and UPLC-qToF-MS-based metabolomics), we could define wine antioxidant metabolome as the sum of molecular antioxidant markers (AM) characterized by their radical scavenging (AM-RS) and nucleophilic (AM-Nu) properties. The impact of on-lees barrel aging of chardonnay wines on the antioxidant metabolome was studied for two consecutive vintages. The identification of wines’ antioxidant metabolome allows for a detailed understanding of the transient chemical interplays involved in the antioxidant chemistry associated with well-known antioxidants and opens an avenue towards personalized winemaking. The present study gathers for the first time the dynamics of wines’ antioxidant metabolome during on-lees aging. Monitoring the variations of the wine antioxidant metabolome can provide an avenue to better control the winemaking process using the knowledge of how to optimize the wine aging potential.

## 1. Introduction

In a context of societal concern about wine preservation, along with the search for environmentally friendly production characterized by low input and organic-like management, innovation for economical breakthroughs relies on the unprecedented understanding of the complex mechanisms involved in the chemistry of preservatives, among which sulfites have played a major role for centuries. Wines’ stability markedly depends on the stability of each component of the system (microbial and chemical); moreover, destabilization of one component may induce destabilization of another. In situations where microbial stability is achieved through various chemical and/or physical processes, the main issue remains to be the chemical stability against oxidation. This is particularly true for wines whose high added value relies on their aging potential. Indeed, the reputation of great wines is synonymous with the stability of their aroma flavor as young wines while developing specific varietal nuances during aging. In the particular case of dry white wines, oxidative stability relates to the actual worldwide problem of premature oxidation, which concerns cellar-worthy white wines within just a few years of the vintage. Today, more than a decade after the problem first emerged, its scope remains wide, and the causes are poorly understood. Recent figures indeed suggest that up to 4% of tasted wines can be rejected as oxidized by consumers, thus having a potential economic impact of tens of millions of euros for the wine industry.

Up to now, sulfites remain common preservatives used for antimicrobial and/or antioxidant (browning) stability in fruits, vegetables, and processed foods [1]. However, due to existing health concerns derived from the consumption of high concentrations of sulfites, there is a current trend to limit its use during food processing and to look for alternatives [2]. So far, none of the attempts to find alternatives for preserving wine from autoxidation have succeeded. Glutathione (*γ*-l-glutamyl-l-cysteinylglycine, GSH) is considered a key natural compound that can manage food and wine stability under oxidative aging conditions [3]. Studies dealing with wine oxidation were in their majority focused on the description of antioxidant effects of sulfites or GSH via monitoring the evolution of potent volatile oxidation markers without giving any insights into the mechanisms controlling their formation [4].

In order to gain control of the wine oxidation mechanism, we need a solid fundamental understanding of the fate of major oxidant compounds (i.e., radical species, quinones) produced during the cascade of oxidation reactions. Ideally, researchers could approach the control of off-odor formation in addition to the traditional targeted compound analysis, by searching for native chemical regulators of oxidation mechanism like GSH [5]. It appears that a number of natural wine constituents may be participating in the oxidation reactions, especially in the secondary reactions which are yet unknown [6]. Understanding what these metabolites are, as well as looking at substances that have antioxidant properties in other fermented beverages, will allow us to directly assess wines’ natural resistance against oxidation. This alone will be very valuable in estimating the shelf life and understanding aging potential. By applying the combination of powerful and modern analytical approaches (EPR, FTICR-MS-based metabolomics), recent studies from our laboratory established a molecular database referred to as the antioxidant metabolome, related to white wines’ oxidative stability expressed from the very beginning of the winemaking process [7]. Moreover, considering the importance of peptides and amino acids for the wine antioxidant metabolome, our research team proposed [8] a one-step 4-methyl-1,2-benzoquinone derivatization method to enhance thiol ionization efficiency and give a better screening of specific S- N-containing functional compounds as part of the white wines’ antioxidant metabolome. UHPLC-qTOF-MS analysis of up to 92 white wines from different cultivars and vintages (Chardonnay, Sauvignon, and Semillon) allowed us to putatively identify up to 141 wine-relevant nucleophiles, representing the nucleophilic molecular fraction of white wines’ antioxidant metabolome. Our point of view, following the variations of wines’ antioxidant metabolome as defined above, could provide an avenue to better control the winemaking process using the knowledge of how to optimize wine oxidation potential.

To that purpose, the present study, for the first time, gathers complementary and synoptic expertise on the impact of on-lees aging in oak barrels on wines’ antioxidant metabolome. Special attention was paid to the reactional activity of sulfur-containing antioxidant compounds in order to understand the chemical pathways involved in their consumption.

## 2. Materials and Methods

### 2.1. Chemicals

MS grade acetonitrile was obtained from Biosolve (Dieuze, France), formic acid (MS grade) from Acros Organic (Morris Plains, NJ, USA), ethanol from Fisher Chemical (Loughborough, UK), Tuning Mix from Agilent Technologies (Santa Clara, CA, USA), 4-methyl-catechol (4MeC), Amberlyst A-26(OH) ion-exchange resin, periodic acid, 1,1-Diphenyl-2-picrylhydrazyl radical (DPPH), citric acid, sodium phosphate dibasic were purchased from Sigma Aldrich (St. Louis, MO, USA). Methanol (99.8%) was purchased from Chemlab. Ultrapure water comes from a Milli-Q system (Merck, Darmstadt, Germany).

### 2.2. Wine Samples

Chardonnay wines from 14 vineyard blocks from the Burgundy region (northeast France) from 2016 and 2017 vintages were aged on lees in oak barrels for 12 months. Samples from the 2016 vintage were analyzed after 12 months of aging, while samples from the 2017 vintage were analyzed during different steps of the winemaking process (after alcoholic fermentation (0 months), after 4 months, and after 12 months of aging). Oak barrels were all 2 years old in order to limit wood-derived ellagitannin extraction. A detailed description of wine samples is given in Table S1.

### 2.3. Wines Antioxidant Capacity by DPPH Analysis

Antioxidant capacity measurements have been conducted using the optimized protocol for white wines proposed by Romanet et al., 2019 [9]. For this, we used a 25 g/L DPPH solution in methanol—citrate (0.1 M)/phosphate (0.2 M) buffer (3/2 (*v*/*v*) and pH 3.6). Increasing volumes of wines were analyzed (0 µL < V < 100 µL) into 3.9 mL of DPPH solution to create calibration curves. To prevent oxidation, samples were prepared in a glove box under nitrogen atmosphere. Samples were incubated for 4 h in dark at room temperature before being analyzed by spectrophotometry at 525 nm. Before analysis, free SO_2_ was removed by degassing wines using CO_2_ bubbling [10].

### 2.4. Ultra-High-Performance Liquid Chromatography Coupled to a Quadrupole-Time of Flight Mass Spectrometer Analysis

Analyses were performed using ultra-high-pressure liquid chromatography (Dionex Ultimate 3000, Thermo Fisher Scientific, Waltham, MA, USA) coupled to a MaXis plus ESI-qToF mass spectrometer (Bruker, Bremen, Germany). Non-polar compounds were analyzed in reverse phase using Acquity BEH C18 1.7 µm, 100 × 2.1 mm column (Waters, Guyancourt, France). Mobile phase was A) acidified water (0.1% (*v*/*v*) of formic acid) and B) acidified (0.1% (*v*/*v*) of formic acid) water/acetonitrile (5/95 *v*/*v*). The temperature of elution was 40 °C, and the column gradient as follows: 0–1.10 min 5% (*v*/*v*) of eluent B and 95% of eluent B at 6.40 min. The flow was 400 µL/min. The pressure of the nebulizer was 2 bars and 10 L/min for nitrogen dry gas flow. Ionization was realized in electrospray in positive or negative ion mode. Parameters of ions transfer were 500 V for end plate offset and 4500 V for capillary voltage. Injection of 4 times diluted calibrant ESI-L Low concentration Tuning Mix (Agilent, Les Ulis, France) at the beginning of each run, allowed to recalibrate spectra. Before each batch analysis, the mass spectrometer was calibrated using undiluted Tuning Mix in enhanced quadratic mode (errors < 0.5 ppm). The mass range was between 100 and 1000 *m*/*z*. To verify the stability of the UHPLC-qTOF-MS system, quality controls (mix of all samples) were analyzed at the beginning, at the end, and every 10 samples during the batch. Samples were analyzed randomly.

### 2.5. Analysis of Wine-Relevant Nucleophiles by 4-Methyl-Benzoquinone Probing

Wine-relevant nucleophiles were analyzed using the protocol proposed by Romanet et al., 2020 [8]. Wine samples, without pH adjustment, were probed using 4-methyl-benzoquinone (4Me-Q) prepared in acetonitrile as proposed by Nikolantonaki & Waterhouse, 2012 [11]. 1 mmol/L of 4Me-Q was added to 1 mL of wines after 30 min incubation in dark, 1 mmol/L SO_2_ was added to quench the reaction. Blank samples were prepared each time by adding acetonitrile and sulfites in equal concentrations as in the derivatized samples. Derivatized samples were analyzed by an UHPLC-qTOF-MS system within 24 h, as described in Section 2.4.

### 2.6. Data Mining

UHPLC-qTOF-MS features (couple *m*/*z*, retention time) were filtered according to S/N (>30) and absolute intensity (>1000). Before features extraction, the spectral background noise was removed. Features were aligned using a homemade R script, with *m*/*z* and retention time tolerance lower than 10 ppm and 0.3 min, respectively. Spearman correlation between features and EC_20_ was calculated using R script to isolate correlated features (|r| > 0.6 and *p*-Value < 0.01). To isolate nucleophilic compounds, partial least squares discriminant analyses (PLS-DA) were performed with Simca (Umetrics) to determine VIP scores for each feature. Using a Matlab script (R2015a), the Wilcoxon correlation was calculated. Features were filtrated using VIP > 1 and *p*-Value < 0.01, allowing to isolate native and derivatized nucleophilic compounds [8]. The elementary composition of the filtrated compounds was determined using isotopic profiles with DataAnalysis (v. 4,3, Bruker, Mannheim, Germany). Annotation was realized using online databases KEGG, Metlin, and the online tool Oligonet [12,13,14]. Targeted research of reaction products from wine-relevant nucleophiles was realized using a Matlab script, based on the reaction: $AOH+Nu\;\to P+2H^{+}$, where *AOH* is a phenolic compound, *Nu* is a nucleophilic compound (AM-Nu), and *P* is their reaction product detected in the wine. Ten phenolic compounds have been selected to manage the reaction (gallic acid, caffeic acid, coumaric acid, ferulic acid, caftaric acid, and coutaric acid, hydroxytyrosol, tyrosol, catechin, and quercetin). Figures were plotted using Origin Pro 8 software (Originlab Corporation, Wellesley Hills, MA, USA), Matlab, and Simca.

## 3. Results and Discussion

### 3.1. Evolution of Wines Antioxidant Capacity during On-Lees Barrel Aging

**Figure 1:**
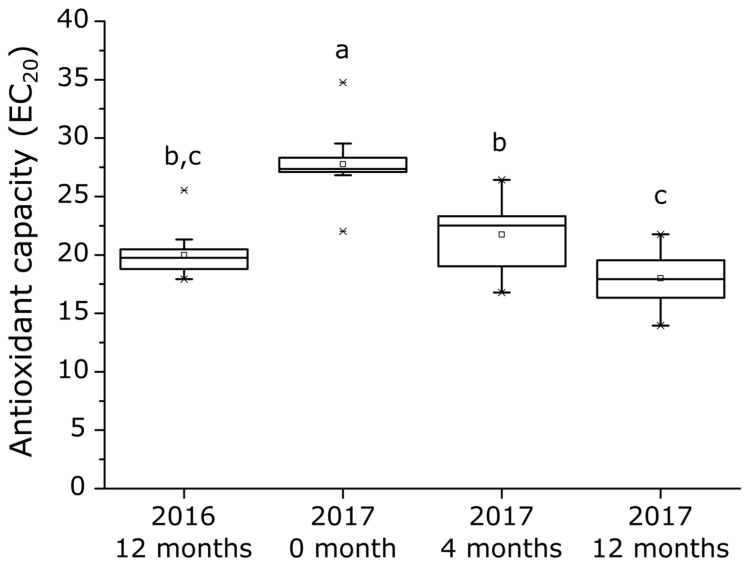
Antioxidant capacity of wines (EC_20_) according to the vintage (2016, n = 14, and 2017, n = 42) and the on-lees barrel aging duration. Box plots represent median, first, and third quartile (Q1, Q3); whiskers represent 1.5 times interquartile ranges (Q1–Q3); squares visualize mean and cross minimal and maximal values. Different letters indicate significantly different groups determined using ANOVA and Tukey test (*p*-Value < 0.05).

White wines originating from 14 vineyard blocks harvested in 2016 and 2017, were aged on lees for 12 months. Samples from the 2016 vintage were analyzed at 12 months (n = 14), while samples from the 2017 vintage were analyzed during different steps of the winemaking process (after alcoholic fermentation (0 months; n = 14), at 4 months (n = 14), and at 12 months (n = 14)). Wines’ antioxidant capacity was evaluated using the DPPH protocol optimized for white wines and expressed as EC_20_ [9]. Figure 1 shows the evolution of the mean EC_20_ values during barrel aging on lees for both 2016 and 2017 vintages. Box plots allow visualizing the distribution of EC_20_ values related to the vineyard block, vintage, and aging time on lees. The antioxidant capacity of wines significantly (Anova *p*-Value < 0.05) increases during on-lees barrel aging, where the EC_20_ values vary from 27.8 ± 2.9 (2017 at 0 months) to 17.9 ± 2.4 (2017 at 12 months). It is worth mentioning that no vintage effect was observed on wines’ antioxidant capacity at 12 months. Indeed, on-lees aging is an important winemaking step during which wines become naturally enriched in antioxidant compounds due to yeast autolysis [15,16]. Autolysis occurs from the 6th month post-alcoholic fermentation and depends on the wine’s carbon dioxide concentration, alcohol level, pH, and temperature [15,16]. Optimum conditions are 45 °C and pH 5.0, which are very different from classical wine conditions [17].

During autolysis, internal proteases act to hydrolyze lysosomal and cytoplasmic membranes, and irreversible degradation of cell wall components like glucans and mannoproteins increases cell wall porosity facilitating the release of degraded constituents into the wine [17]. Yeast-derived compounds typically include cytoplasmic (amino acids, peptides, fatty acids, nucleotides) and cell wall materials (proteins, mannoproteins, and polysaccharides), and with grape-derived compounds, they contribute to wines’ intrinsic antioxidant metabolome related to their oxidative stability. Moreover, today’s scientific knowledge does not allow for the discrimination among the grape- and yeast-derived molecular fractions that compose the antioxidant metabolome of wines.

### 3.2. Definition of Wine Antioxidant Metabolome

**Figure 2:**
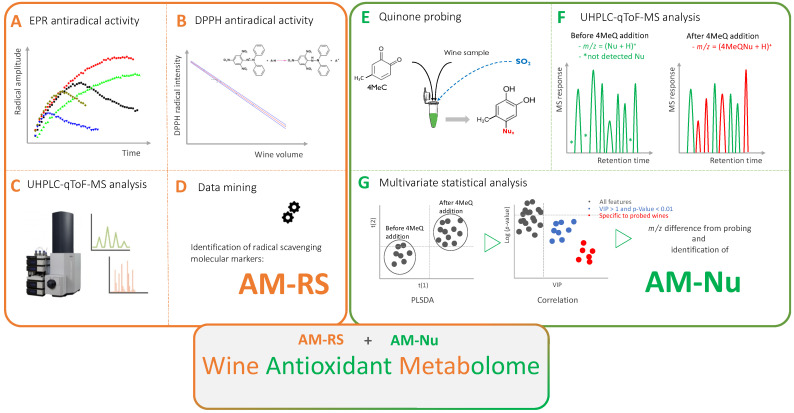
Schematic representation of wine antioxidant metabolome (AM) composed of antiradical scavenging (AM-RS) and nucleophilic (AM-Nu) related molecular markers. AM-RS molecular markers where isolated by applying the combination of powerful and modern analytical approaches: (**A**) EPR antiradical activity, (**B**) DPPH antiradical activity, (**C**) UHPLC-q-Tof-MS analysis and (**D**) data manning. AM-Nu molecular markers where isolated after: (**E**) one step derivatization step using the 4-methyl-1,2-benzoquinone (quinone) as a electrophilic molecular probe, (**F**) the mass difference from the quinone reagent used for identification of wines relevant nucleophiles based on UHPLC-qToF-MS screening of unknown nucleophiles and, (**G**) data mining based on multivariate statistical analysis.

White wines’ antioxidant metabolome is the sum of molecular antioxidant markers characterized by their radical scavenging (AM-RS) and nucleophilic (AM-Nu) properties (Figure 2). AM-RS currently counts up to 380 features (couple ions *m*/*z*, retention time) gathered in an in-lab database created after the analysis of 287 wines where correlations between their antiradical capacity and their molecular profile were established [7]. On the other hand, AM-Nu is an in-lab database currently listing 141 wine-relevant nucleophiles (couple ions *m*/*z*, retention time) identified after direct one-step quinone probing of up to 92 white wines from different cultivars and vintages [8].

### 3.3. Dynamics of Wine Antioxidant Metabolome during On-Lees Barrel Aging

#### 3.3.1. Dynamics of AM-RS

**Figure 3:**
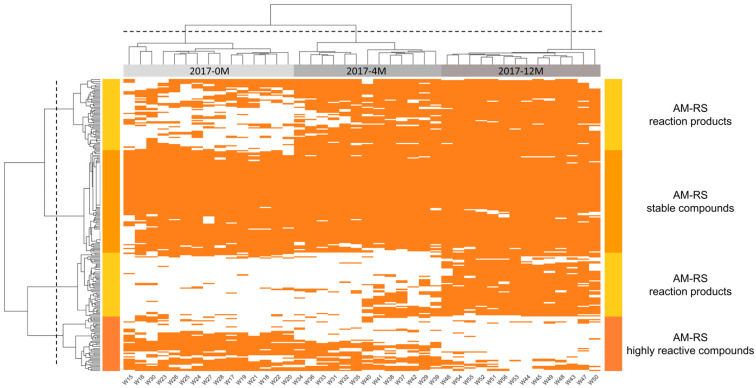
Heatmap of AM-RS dynamic molecular compositions during barrel aging. White areas represent undetected compounds, and orange areas represent detected compounds. Hierarchical clustering analysis allows for the discrimination of three groups of AM-RS compounds: stable compounds, highly reactive compounds, and reaction products.

To provide advanced and yet unraveled scientific knowledge about the antioxidant chemistry associated with wines’ oxidative stability during barrel aging, the dynamic behavior of wines’ antioxidant metabolome was monitored by UHPLC-qToF-MS. In all 56 studied wines, 242 AM-RS features were detected (Table S2). Comparison of the presence or absence of AM-RS molecular markers allows for the discrimination of wines according to on-lees barrel aging duration (Figure 3). It is worth mentioning that among analyzed barrels, W30 showed no significant evolution in its antioxidant capacity and molecular profile at 4 months compared with those from 0 months. W30 represents a sample aged in a double-size barrel (500 L), where we can expect slower wood and lees/wine interaction related to the higher ratio volume/surface. This result confirms empirical observations of winemakers, explaining why they usually use bigger vessels to slow the aging process. However, no significant differences were observed at 12 months for W30, suggesting that the duration of barrel aging has a greater impact on wine composition than the size of the vessel.

The heatmap representation of the dynamic behavior of AM-RS shows that different clusters of features allow for wine discrimination against barrel aging (Figure 3). We also observed three concomitant and distinctive groups of features related to the global increase in wines’ antioxidant capacity. First, 22% of total AM-RS presents a stable fraction of the global antioxidant metabolome. Indeed, this major group of AM-RS stable compounds comprises the molecular buffer of the wines chemical environment, which is related to their antioxidant capacity. Second, we observed the group of decreasing compounds (12% of total AM-RS), which, in our point of view, are highly reactive compounds involved in transient chemical interplays of the antioxidant chemistry associated with wines’ oxidative stability. Third, the group of reactional products (30% of total AM-RS) related to the compounds formed at the late stages of barrel aging (4–12 months).

**Figure 4:**
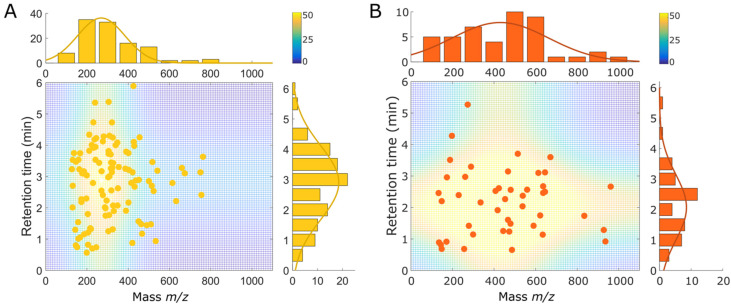
Distribution of AM-RS (**A**) reactional products and (**B**) highly reactive compounds according to their *m*/*z* (Da) and retention time (min). Gaussian distributions have been calculated using $z_{1}=a_{1}*exp\left(-\frac{{\left(x-b_{1}\right)}^{2}}{c2}\right)$ for *m*/*z* and $z_{2}=a_{2}*exp\left(-\frac{{\left(y-b_{2}\right)}^{2}}{c_{2}2}\right)$ for retention time. Surface plot represents the theoretical number of compounds determined by $z=z_{1}+z_{2}$.

In order to better describe the chemical diversity of reactional products and highly reactive AM-RS markers, the distribution according to their *m*/*z* and retention time is presented in Figure 4. AM-RS reaction products come from two distinct branches from the HCA (Figure 3) due to the differences on their retention time monitored by UHPLC-qTof-MS during barrel aging. The Mann–Whitney test on these two branches showed no significant differences (*p*-Value < 0.1) according to their *m*/*z* and retention times. AM-RS reaction products appeared in their majority at lower apparent masses (210 Da < *m*/*z* < 406 Da) for the first and third quartiles (Q1 and Q3) and retention times (Q1 = 2.0 min and Q3 = 3.7 min) than AM-RS highly reactive compounds, presenting higher molecular weights (Q1 = 261 Da and Q3 = 613 Da) and being less polar, thus eluted earlier (Q1 = 1.3 min and Q3 = 2.7 min) (Figure 4A,B). These results based on variations in molecular weight and hydrophobicity show clear differences in the chemical diversity of different AM-RS-related molecular fractions.

To better understand and monitor wines’ antioxidant chemistry through the dynamic behavior of AM-RS, annotations using online databases (Metlin and Oligonet) have been realized to enhance information about the chemical diversity of AM-RS. In addition, the chemical formulae of annotated compounds were determined using isotopic profiles. Sixty-four out of one hundred and twelve AM-RS reactional products were annotated as CHO (n = 22) and CHON (n = 36) compounds (Table S2). Three of them could putatively be assigned to coumarin derivates, C_9_H_6_O_2_ (146.0368 Da, RT = 3.5 min), C_9_H_6_O_3_ (162.0361 Da, RT = 3.2 min), C_10_H_8_O_3_ (176.0473 Da, RT = 3.1 min). These ions could be tentatively identified as coumarin, chromone or phenyl-propiolic acid for C_9_H_6_O_2_, hydroxycoumarin, umbelliferone for C_9_H_6_O_3_ and herniarin, methoxy-chromone, methyl-umbelliferone, or hydroxy-decene-triynoic acid for C_10_H_8_O_3_. Coumarin (C_9_H_6_O_2_), umbelliferone (C_9_H_6_O_3_), and methyl-umbelliferone (C_10_H_8_O_3_) are oak-wood-derived compounds known for their high antioxidant capacity [18,19]. From the same perspective, C_9_H_10_O_3_ (166.0224 Da, RT = 3.8 min) and C_9_H_10_O_5_ (198.0528 Da, RT = 3.0 min) could putatively correspond to phloretic acid or ethyl vanillin and syringic acid or vanillylmandelic acid, respectively [20,21,22,23]. In addition, among the CHO AM-RS reactional products we could annotate C_9_H_14_O_6_ (218.0790 Da, RT = 1.3 min) reported in the literature as triacetin, an inactivate dry yeast derivative [24]. Concerning CHON-containing AM-RS reactional products, we could annotate 15 peptides (i.e., Gly-Pro C_7_H_12_N_2_O_3_ (172.0848 Da, RT = 2.3 min), Glu-Pro C_10_H_16_N_2_O_5_ (244.1059 Da, RT = 0.7 min), C_12_H_23_N_3_O_5_ (289.1638 Da, RT = 1.8 min), C_34_H_55_N_9_O_10_ (749.4072 Da, RT = 3.1 min), C_18_H_32_N_4_O_6_ (400.2321 Da, RT = 2.2 min)), amino acids (i.e., tryptophane C_11_H_12_N_2_O_2_ (204.0899 Da, RT = 1.2 min), and nucleoside derivatives (i.e., guanine C_5_H_5_N_5_O (151.0494 Da, RT = 0.7 min) originating possibly from yeast autolysis [15,25,26,27]. Zheng et al., 2016 [28] have shown that tryptophane has an important antioxidant capacity based on the ABTS and the ORAC assays confirming its potential role in wines’ oxidative stability.

Thirty-one out of forty-five decreasing AM-RS highly reactive compounds were essentially annotated as CHON, being peptides for their majority (n = 12) (Table S2). Peptides are highly reactive compounds, acting as sacrificial nucleophiles to protect wines against chemical oxidation. In order to go deeper into their characterization and reactivity, a complementary UHPLC-qToF-MS probing analysis was run and the detected compounds reported as wines AM-Nu molecular fractions. Sixty-four out of eighty-five AM-RS stable compounds were annotated in their majority (n = 39) as CHON representing compounds associated to peptides (i.e., Pro-Leu C_11_H_20_N_2_O_3_ (228.1473 Da, RT = 1.5 min) and Val-Phe C_14_H_20_N_2_O_3_ (264.1471 Da, RT = 3.1 min)) and amino acids (i.e., Phe C_9_H_11_NO_2_ (165.0789 Da, RT = 1.1 min)). Fifteen were CHO compounds, likely comprising phenolic compounds such as caftaric acid (C_13_H_12_O_9_, 313.0482 Da, RT = 1.4 min), prenyl caffeate (C_14_H_16_O_4_, 248.1045 Da, RT = 3.1 min), and shikimic acid (C_7_H_10_O_5_, 174.0528 Da, RT = 0.8 min) known for their antioxidant properties.

#### 3.3.2. Dynamics of AM-Nu

**Figure 5:**
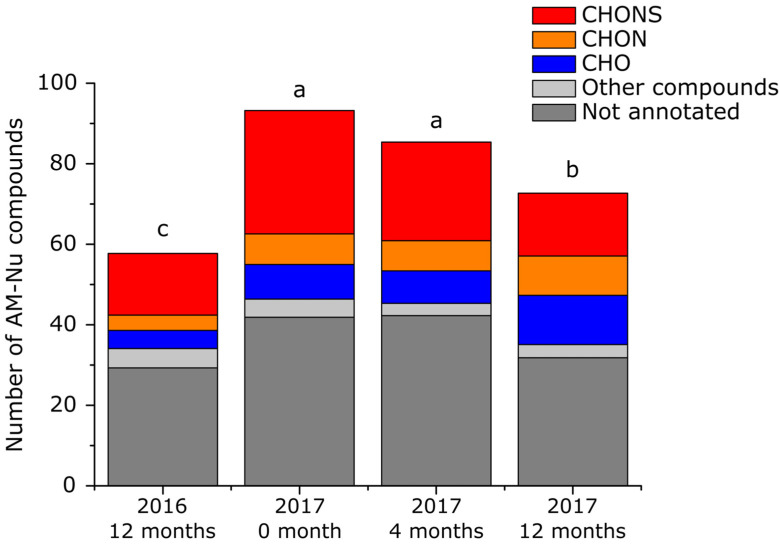
Number of nucleophilic compounds isolated during barrel aging. Annotation has been realized using online tools Oligonet [14], and Metlin. Different letters indicate significative differences determined by ANOVA and Tukey HSD test (*p*-Value < 0.05). Other compounds regroup CH, CHN, CHNS, CHOS and CHOP compounds.

From the 358 AM-NU features, 155 were detected, 86 were respectively annotated as CHO (17%), CHON (14%) CHONS (37%) and others (32%) (Table S3). Figure 5 shows a significant diminution on the number of AM-Nu features during barrel aging, in particular CHONS containing compounds. In detail, on average, in wines from the 2017 vintage, 31 ± 5 CHONS compounds were detected at 0 months, 25 ± 7 at 4 months, and 16 ± 6 at 12 months of barrel aging. Among these, amino acids: Cys C_3_H_7_NO_2_S (121.0197 Da) and Homocysteine C_4_H_9_NO_2_S (135.0354 Da), and peptides: Ala-Cys-Glu C_11_H_19_N_3_O_6_S (321.0995 Da), Cys-Leu-Asp C_13_H_23_N_3_O_6_S (349.1308 Da), Ser-Val-Cys C_11_H_21_N_3_O_5_S (307.1202 Da), Gly-Met C_7_H_14_N_2_O_3_S (206.0725 Da), Val-Leu-Met C_16_H_31_N_3_O_4_S (361.2035 Da) were depleted at 12 months. However, GSH C_10_H_17_N_3_O_6_S (307.0838 Da) and Gly-Cys C_5_H_10_N_2_O_3_S (178.0412 Da) showed a certain stability during barrel aging. These results indicate the difference of reactivity between peptides and amino acids during wine aging conditions and introduce the importance of the chemical diversity of AM-Nu in the oxidative stability of wines. On other hand, barrel aging has a lower impact on the number of CHO and CHON of AM-Nu compounds. CHO compounds are essentially condensation reaction products with the molecular probe (4MeQ) that would occur under our experimental conditions. The number of CHO compounds significatively increases during barrel aging (Anova *p*-Value < 0.05), going from n = 9 ± 3 at 0 months to n = 12 ± 2 at 12 months. At the same time, the number of CHON compounds is increasing during barrel aging. Annotations suggest that these CHON compounds are not peptides and further investigation is needed to better explore their structures.

### 3.4. Vintage Effect on Wines AM-RS and AM-Nu Chemical Diversity

The comparison of wine samples at the end of barrel aging, from 2017 and 2016 vintages showed a significant vintage effect on AM-RS and AM-Nu molecular fractions (Figure S1 and Figure 5). The number of AM-RS and AM-Nu molecular markers is significantly (Anova *p*-Value < 0.01) more important in wines from 2017 (n_AM-RS_ = 103 ± 7, n_AM-NU_ = 73 ± 5) compared with those from 2016 (n_AM-RS_ = 168 ± 7, n_AM-NU_ = 58 ± 12). Interestingly, even if the total number of CHONS compounds is not vintage-dependent, a significant difference was detected in the ratio of AM-RS and AM-Nu molecular fractions. Taking into account that the AM-Nu molecular fraction is essentially composed of reactive peptides, we conclude that peptide populations are mainly the molecular fraction that buffers wines’ oxidative stability. From that perspective, future peptidomics-based studies would allow for better winemaking management by understanding the origin and impact of different winemaking steps on the chemical diversity of peptides.

### 3.5. The Fate of AM-RS and AM-Nu Compounds during Barrel Aging

As illustrated above, dynamics of AM-RS and AM-Nu clearly show that wines’ antioxidant metabolome comprises populations of a diversity of chemical compounds that are very reactive during barrel aging. This reactivity allows of wine maturation and chemical stabilization prior to bottling. Numerous authors have already reported studies of the mechanisms involved in wine oxidation, and many of them were targeted phenolic compounds, considered to be primary substrates for chemical oxidation during barrel and bottle aging [6]. Above all, these results emphasize the wine composition dependency on the oxygenation process, where different wines could exhibit different reactivity towards oxygen, thus stressing the need for analytical approaches that can provide comprehensive pictures of subtle mechanistic variations among series of samples [29]. To understand how AM-RS and AM-Nu compounds could be consumed, semi-targeted analysis has been performed by searching putative oxidative products in the studied wines. Thus, based on the reaction: $AOH+Nu\;\to P+2H^{+}$*,* where *AOH* is a phenolic compound, *Nu* is an AM-Nu compound, and *P* is the reaction product detected in the wines, putative reaction products have been isolated. Phenolics compounds have been selected to be the most relevant substrate for dry white wines (gallic acid, caffeic acid, coumaric acid, ferulic acid, caftaric acid, coutaric acid, hydroxytyrosol, tyrosol, catechin, and quercetin).

On this basis, 66 putative reactions could be isolated, producing 47 reaction products significantly impacted by barrel aging (Anova, *p*-Value < 0.05), Table S4. Some of these are formed by consumption of GSH, Cys, or Cys-Gly, which have been detected in CHONS nucleophilic compounds and can explain their consumption (see Table 1).

Indeed, reaction products resulting from the addition of GSH on caffeic acid (*m*/*z* = 485.1100 Da/RT = 2.5 min), coutaric acid (*m*/*z* = 601.1212 Da/RT = 2.6 min), and catechin (*m*/*z* = 595.1469 Da/RT = 1.3 min) were detected. The ion with a mass of 409.0931 Da at 1.4 min could have been detected as catechin—Cys reaction product. Moreover, the dipeptide Gly-Cys conjugates with hydroxy-tyrosol (*m*/*z* = 330.0882 Da/RT = 2.5 min), tyrosol (*m*/*z* = 314.0930 Da/RT = 3.6 min), and caffeic acid (*m*/*z* = 356.0679 Da/RT = 1.4 min) were also identified. In addition, the amino peptides Gly-Met could react with caftaric acid resulting in the formation of a reaction product with *m*/*z* = 516.1046 Da at 2.7 min.

These results allow for the demonstration that sulfur-containing compounds and essentially peptides are the main key sacrificial nucleophiles in white wines under oxidation conditions. However, taking in account the fact that under wine acidic pH = 3–4, the amino peptides are less nucleophilic that the sulfur-containing ones [8], the reactivity of amino peptides cannot be overlooked and need to be better explored.

**Figure 6:**
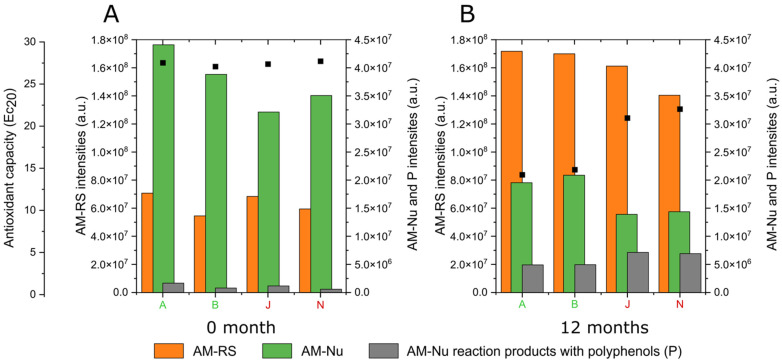
Representation of the sum of intensities of detected ions composing wines antioxidant metabolome with AM-RS (orange), AM-Nu (green), and AM-Nu reaction products with polyphenols (P, grey) and different levels of antioxidant capacity (High or Low EC_20_) (black square) for selected wine samples (A, B, J, N) (**A**) at the beginning and (**B**) at 12 months of barrel aging.

The reactivity of AM-Nu molecular markers with selected polyphenols allows for the first time to elucidate the chemical mechanisms involved in wines’ oxidative stability. Based on that, it is now possible to better describe the transient antioxidant capacity during wine aging. Indeed, we studied the chemical composition of selected wines (A, B, J, N) from the 2017 vintage at 12 months of barrel aging, presenting high (A and B) and low (J and N) antioxidant capacity, with EC_20_ of 14.0 to 21.8, respectively. Figure 6B presents the semi-quantitative analysis of AM-RS, AM-Nu, and *P* related features plotted in correlation with their EC_20_ value for wine samples analyzed in the same batch. It appears that at the end of barrel aging (12 months) wines with high antioxidant capacity are concentrated in AM-RS and AM-Nu molecular markers, while an important fraction of AM-Nu is already consumed and many reactional products with polyphenols are formed. The diversity and the number of *P* (AM-Nu-polyphenols reaction products) provide a complementary information to that given from only the consideration of AM-RS and AM-Nu molecular markers, leading to a better understanding of wines’ oxidative stability during barrel aging. Moreover, it would be interesting to explore if the characterization of wines’ antioxidant metabolome (AM-RS and AM-Nu) just after alcoholic fermentation (0 months) could allow for the prediction of their antioxidant capacity at the end of barrel aging. It appears that only the AM-Nu molecular fraction estimated in freshly made wines is discriminant to wines’ oxidative stability at the end of barrel aging (Figure 6A).

## 4. Conclusions

In this study, the monitoring of 14 wines during the on-lees barrel aging over a period of 12 months during two consecutive vintages (2016 and 2017) allowed for the detailed characterization of the molecular fraction responsible for their oxidative stability. Generally, in all wine samples, the antioxidant capacity estimated by the DPPH essay (EC_20_) is increasing during barrel aging. This result suggests a chemical stabilization of wines during the on-lees aging independently of the vintage. White wines’ antioxidant metabolome, composed of molecular markers with radical scavenging (AM-RS) and nucleophilic (AM-Nu) properties, was profiled during barrel aging. First, it appears that 242 out of 380 AM-RS molecular markers could be detected in all samples, belonging to 3 distinctive subgroups based on their dynamic behavior related to their chemical stability (stable, reactional products, and highly reactive compounds) during barrel aging. On the other hand, 155 out of 358 AM-Nu compounds from our in-lab database are mostly sulfur-containing compounds presenting great nucleophilic character, characterized after the identification of putative reaction products with selected wine-relevant polyphenols. Generally, the antioxidant capacity of wines is increasing during on-lees barrel aging, while an interested correlation was established between the number of AM-Nu related compounds at the beginning of the winemaking process and the wines’ antioxidant capacity at the end of barrel aging.

The discrimination of wines according to their antioxidant capacity, AM-RS, AM-Nu, and putative reaction products with polyphenols (P) composition has shown the importance of cross-checking the information between analyses to take into account the high diversity and complexity of the compounds involved in the antioxidant metabolome of white wines. Indeed, following the variations of wine antioxidant metabolome opens an avenue towards personalized winemaking through first, the categorization of freshly made wine matrices according to their native antioxidant composition and second, the knowledge of how it might be possible to enrich it during the whole process.

## Figures and Tables

**Table 1:** Extract of Table S4 presenting the summary of reactions between targeted phenolic compounds (*AOH*) and AM-Nu molecular markers based on reaction $AOH+Nu\;\to P+2H^{+}$.

*AOH*			AM-Nu			Reaction Product		
Compound	Elementary Formula	Neutral Mass (Da)		Elementary Formula	Neutral Mass (Da)	Elementary Formula	Neutral Mass (Da)	Retention Time (min)
Catechin	C_15_H_14_O_6_	290.0790	Cys	C_3_H_7_NO_2_S	121.0197	C_18_H_19_NO_8_S	409.0831	1.4
Caffeic acid	C_9_H_8_O_4_	180.0423	Gly-Cys	C_5_H_10_N_2_O_3_S	178.0413	C_14_H_16_N_2_O_7_S	356.0679	1.4
Hydroxy-tyrosol	C_8_H_10_O_3_	154.0630	Gly-Cys	C_5_H_10_N_2_O_3_S	178.0413	C_13_H_18_N_2_O_6_S	330.0886	2.5
Tyrosol	C_8_H_10_O_2_	138.0681	Gly-Cys	C_5_H_10_N_2_O_3_S	178.0412	C_13_H_18_N_2_O_5_S	314.0936	3.6
Caffeic acid	C_9_H_8_O_4_	180.0423	GSH	C_10_H_17_N_3_O_6_S	307.0838	C_19_H_23_N_3_O_10_S	485.1104	2.5
Catechin	C_15_H_14_O_6_	290.0790	GSH	C_10_H_17_N_3_O_6_S	307.0838	C_25_H_29_N_3_O_12_S	595.1471	1.3
Coutaric acid	C_13_H_12_O_8_	296.0532	GSH	C_10_H_17_N_3_O_6_S	307.0838	C_23_H_27_N_3_O_14_S	601.1213	2.6
Caftaric acid	C_13_H_12_O_9_	312.0481	Gly-Met	C_7_H_14_N_2_O_3_S	206.0722	C_20_H_24_N_2_O_12_S	516.1047	2.7

## Data Availability

Data is contained within the article and supplementary material.

## Supplementary Materials

The following supporting information can be downloaded at: https://www.mdpi.com/article/10.3390/antiox12020395/s1, Table S1: General description and codes of all wine samples, Table S2: AM-RS compounds and their relative intensities in all wine samples, Table S3: AM-Nu compounds and their relative intensities in all wine samples, Table S4: AM-Nu reaction product with polyphenols and their relative intensities in all wine samples, Figure S1: Comparison of AM-RS compounds at 12 months of aging for 2016 and 2017 vintages.
