# Supplementary material for: White Wine Antioxidant Metabolome: Definition and Dynamic Behavior during Aging on Lees in Oak Barrels
Source: Antioxidants (Basel). 2023 Feb 6;12(2):395. doi: 10.3390/antiox12020395 (PMC9952738; doi:10.3390/antiox12020395)
Supplement: Supplementary file 1 [file antioxidants-12-00395-s001.zip › antioxidants-2167807-supplementary/supplementary data/Figure S1.pdf]

White wines antioxidant metabolome: Definition and dynamic behavior during aging on lees in oak barrel aging

Rémy ROMANET, Régis D. GOUGEON and Maria NIKOLANTONAKI*

Université de Bourgogne, Institut Agro Dijon, PAM UMR A 02.102, Institut Universitaire de la Vigne et du Vin – Jules Guyot, F-21000 Dijon, France

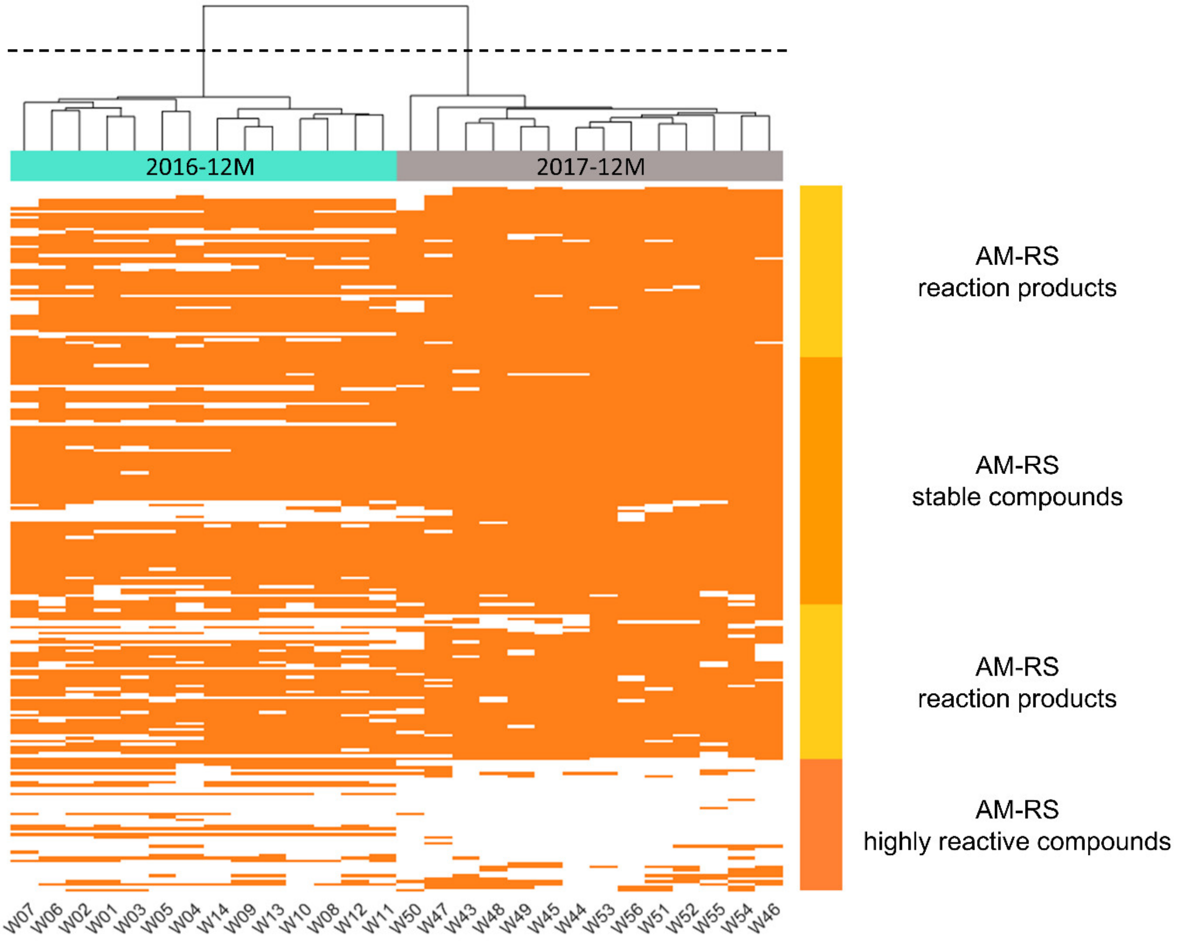

Figure S1. Heatmap of AM-RS comparing wines from vintage 2016 and 2017 after 12 month of barrel aging. White areas represent undetected compounds, orange areas represent detected compounds. Discrimination of three groups of AM-RS compounds: stable compounds, highly reactive compounds and reaction products is based on Hierarchical clustering analysis obtained for AM-RS dynamic behavior of 2017 vintage (Figure 3).
